# Painless Labour

**Published:** 1900-09-22

**Authors:** 


### HOSPITAL CLINICS AND MEDICAL PROGRESS.

### PAINLESS LABOUR.

WE referred some months ago to a new method of obtaining insensibility to pain by means of injecting a solution of cocaine into the subarachnoid space in the lumbar spine, and at the International Medical Congress in Paris the subject came up again. Dr. Severeanu, of Bucharest, reported 70 cases in which he had induced surgical anæsthesia in this way; and M. Tuffier said that he had performed 125 operations, among which were 58 laparotomies, after anæsthesia produced by this method; and several other speakers reported similar cases. One of them, M. Racoviceanu-Pitesci, of Bucharest, said that in a large proportion of the cases symptoms of slight intoxication followed the use of the cocaine, sometimes lasting four or five days. In three of the patients these symptoms were so severe as to necessitate artificial respiration and the use of subcutaneous injections of ether; and he further stated that he knew of two cases in Roumania in which death had followed cocaine anæsthesia induced by lumbar puncture. He did not think, therefore, this method could replace chloroform anæsthesia. Even M. Tuffier gave as among the phenomena following the injection—nausea and vomiting when too large a dose has been given, pallor, respiratory distress, and frequency of pulse; and he said that more or less violent headache, lasting from 15 to 24 hours, was almost always observed. We mention these points to show that this method of producing anæsthesia is not without its drawbacks and even dangers, lest what we are about to say in regard to the use of this same proceeding in labour should lead men into difficulties unawares. Dr. S. Marks,[1] of New York, stimulated as he says by the results obtained by M. Tuffier, has utilised the same plan for the purpose of mitigating, if not entirely allaying, the pains of labour. He properly says that the cases are too few as yet to justify absolute deductions, yet, he adds, they are sufficient to warrant him in stating "that in the lumbar cocainisation we have a method which is of the greatest value in producing analgesia, which checks almost entirely the pains of labour without, so far as personal experience goes, the least danger to mother or child." He goes on to describe the effect of the method in the following terms: "It is very astonishing and awe-inspiring to those of us who have seen many labours, and heard the agonising and maniacal shrieks of these poor women, to see the 'parturient' under the influence of cocaine lie quietly in bed, feeling some indescribable sensation, but without pain, bearing down when told to, and giving birth to her child without her knowledge, and cognisant only of the fact when the first cry of the new-born is heard." Probably the degree of analgesia which he aimed at was much less than that obtained by the surgeons already mentioned who employed the process in surgical work, for he says, "Pain, if present, was certainly bearable, and infinitely lessened under cocaine as compared to such manipulations done without local or general narcosis." The operation of lumbar puncture for cocainising purposes is practically the same as when it is undertaken for purposes of diagnosis, and the same precautions in regard to ensuring complete asepsis in every part of the proceeding must be scrupulously attended to. The following is the process as described by Dr. Stone, house surgeon at the New York Maternity Hospital: "The patient's back, from the coccyx to the middle of the dorsal vertebræ, is thoroughly scrubbed with tincture of green soap and alcohol and ether. This is followed by a saturated solution of permanganate of potash which is removed by a supersaturated oxalic-acid solution. The entire area is then covered with sterile towels. A needle about ten cm. long is employed with a metal hypodermic syringe, both of which are boiled ten minutes. The patient is placed on the side with arched back. The thumb of the left hand is placed on the spinous process of the fourth lumbar vertebra. (This point may readily be found by locating the deep depression between the spine of the last or fifth lumbar and first sacral, the posterior landmark of the external conjugate, or, in very fat women, a line drawn joining the highest points of the crista ilii will pass over the centre of the fourth lumbar vertebra, and is a reliable guide.) The needle is inserted half an inch in front of and just outside the edge of the thumb, at an angle of about 165 deg. The direction of the needle is from below upward and without inward. If the point strikes the lamina it is to be moved gently up or down until the space between the vertebræ is felt. The puncture may be made either between the third and fourth or fourth and fifth vertebræ. The point is then pushed slowly and gently onward until the spinal fluid is seen running out. Ten minims of a cocaine solution, representing gr. 1/6, are now injected and the needle withdrawn. This is all that is necessary." Yes, that is all; but in view of what was stated in Paris as to this method, we would advise our readers to wait awhile before committing themselves to any such proceeding.

[1] New York Med. News.

